# Paternal investment and low birth weight – The mediating role of parity

**DOI:** 10.1371/journal.pone.0210715

**Published:** 2019-01-24

**Authors:** Anna Merklinger-Gruchala, Grazyna Jasienska, Maria Kapiszewska

**Affiliations:** 1 Faculty of Medicine and Health Sciences, Andrzej Frycz Modrzewski Krakow University; Krakow, Poland; 2 Department of Environmental Health, Faculty of Health Sciences, Jagiellonian University Medical College, Krakow, Poland; University of Missouri Columbia, UNITED STATES

## Abstract

According to life-history theory, paternal investment affects the well-being of offspring. We hypothesized that environmental stress caused by a lack of paternal investment may diminish maternal resource allocation during pregnancy, especially for women who already have dependent children. Our study was conducted on a representative group of more than 80,500 singleton, live-born, full-term infants born in Krakow, Poland in the period 1995–2009. Birth data were obtained from the birth registry. We found that missing data about fathers (a proxy measure of low paternal investment) was associated with higher probability of multiparous mothers giving birth to low-birth-weight infants (1.48; 95% CI 1.05–2.08), but this was not the case with primiparous mothers (1.19; 95% CI 0.89–1.59). The statistically significant synergistic effect between parity and paternal investment was found (Synergy Factor = 2.12; 95% CI 1.47–3.05, p<0.001). These findings suggest that in situations of low paternal investment, multiparous mothers face trade-offs between investing in existing versus unborn children, therefore investment in the latter is lower. Such a strategy may benefit maternal fitness due to investment in older children, who have higher reproductive value.

## Introduction

The concept of parental investment [1–2] suggests that all parents’ actions contribute to the well-being of offspring and thus to their reproductive success. Due to the limitations of parental abilities and the effort required to successfully rear each offspring, investment in a particular child is often associated with a compromise in investment in other children (present or future). Because reproductive success can be understood as the number of offspring who survive to reproduction, each individual child has a different reproductive value for the parent [1–2]: offspring who are likely to provide a higher reproductive return of investment are favoured by parents [3]. Reproductive value increases with the age of a child and thus, everything else being equal, younger offspring are less valued than older ones [4]. Maternal investment begins in utero; thus, lower birth weight is one of the main indicators of lower maternal investment during pregnancy [5]. Larger body size is beneficial for offspring, but it is often associated with costs for the mother due to greater metabolic burden during pregnancy [6]. Given the high metabolic costs of pregnancy, lower investment in current offspring may occur during periods of environmental constraints [7–8]. Investment in a current pregnancy might decrease in response to environmental psychosocial stress and reproductive potential may theoretically be reserved for the future, when conditions might become more favourable [5]. The relations between maternal prenatal stress and lower birth size have been documented [9–10] and interpreted in terms of the plasticity of human life history strategy [11]. A large body of evidence suggests that the trade-offs between investment in current vs. future reproduction may be affected by the presence or absence of the commitment (investment, involvement, support) a woman receives from her partner [7, 12]. The absence or low level of a partner’s involvement implies that a woman faces trade-offs in terms of her own future life course (i.e. possible future reproduction), education prospects and the ability to marry well [13]. If a partner is unable or unwilling to provide critical resources for child-rearing, the mother’s most likely strategy would be to minimize her investment in the current pregnancy in order to minimize the costs of the current reproduction [14]. It is well documented that low paternal investment is associated with lower physical well-being of children and higher mortality in infancy [see 12 for review]. However, during pregnancy less paternal support, which is expressed mainly via emotional involvement and financial provisioning, may negatively affect perinatal outcomes [15–18]. These findings are in line with the results of many studies that suggest that being unmarried (vs. married) or in a non-cohabiting romantic relationship with the father (vs. cohabiting with the father) is associated with an increased risk of adverse pregnancy outcomes, such as a low birth weight [18–19].

Furthermore, maternal parity (i.e. the number of previous births), viewed as a rough proxy of the costs of reproduction [8, 20], is clearly an important factor that affects the risk of having a low birth weight (LBW) infant [21–23]. The high costs of reproduction for a woman living in an energy-poor environment may lead to deterioration of her health, i.e. “maternal depletion syndrome” [24]. Therefore, we expect that a pregnant woman who does not have the commitment of her partner as she approaches child birth has a higher risk of delivering an LBW infant, especially if it is not her first child. Such circumstances (multiparity with no commitment from the partner) are especially costly for a woman in terms of her lifetime reproductive strategy.

The association between paternal investment and birth outcomes has been explored with the use of birth registry data. For example, establishment of paternity for children of unmarried mothers was used as a proxy for both high paternity confidence and male willingness to commit to paternal investment; it was also found to predict birth outcomes, including low birth weight [25]. Missing fathers' names in birth registry data can also be treated as a surrogate measure of a lack of paternal involvement and was found to be a risk factor of infant mortality and low birth weight [26]. Similar proxy indicators were also used in other studies based on birth certificates, including missing information about partner’s age or ethnicity [27–28]. In most of these studies (but not all, see, for example [28]) a lack of paternal involvement was associated with worse maternal well-being or birth outcomes [26–27, 29]. Missing paternal data as a proxy indicator of paternal involvement was treated as a convenient tool for identifying populations with a high risk of adverse pregnancy outcomes. However, the interactions between maternal parity and missing paternal data have not been examined so far.

## Material and methods

According to Polish legislation, one way of determining paternity is to presume that the mother’s husband is her child’s father. Therefore, the name of the mother’s husband is listed on a child’s birth record. For unmarried parents, an unmarried father must sign an acknowledgment or have paternity established in court in order to have his data on the birth certificate [30]. The presence of the father's data (such as his age, education and employment) on a birth record suggests that the acknowledged or court-established father is more likely to be in a close relationship with the mother, perhaps even living with her. Such fathers might also be involved in the support and care of the pregnant woman. On the other hand, the lack of a father's data on the birth record may indicate that the mother does not know the father or is unwilling to identify him. Missing paternal data on birth records was a variable of primary interest in our study and we used it as a surrogate measure of low paternal investment. This data was considered absent from the birth record if all fields provided for the father's data, i.e. his age, education and employment were blank.

The study group was restricted to singleton live-born infants to remove the effect of multiple gestations and stillbirths. We included infants born after gestations of 37 and 41 weeks from the 14-year period from October 1st, 1995 to December 2009 to mothers whose residence at the time of the infant’s birth was the city of Krakow. We obtained anonymized data from the birth registry (Central Statistical Office in Poland). Data included month and year of birth, birth weight (in grams), infant’s sex, maternal age (in years), gestational age (in weeks), parity, maternal education (primary, lower secondary, basic vocational, upper general or specialized secondary and academic education), maternal employment status (employed vs. not employed) and maternal marital status (married vs. not married, i.e. single, widowed, divorced or separated). Paternal data, if present, included age (in years), education (primary, lower secondary, basic vocational, upper general or specialized secondary and academic education) and employment status (employed vs. not employed). Low birth weight (LBW) was defined as birth weight below 2,500 g; all infants with birth weight ≥ 2,500g were classified as having normal birth weight (NBW).

Of 88,474 singleton births, we excluded N = 4,648 born before 37 weeks of gestation, N = 3,141 born later than 41 weeks of gestation, and N = 132 stillbirths. These exclusions left N = 80,553 eligible births, and among them N = 1615 (2.0%) LBW newborns.

### Statistical analysis

In order to identify potential confounders, the distribution of several known risk factors for LBW across different exposure categories was examined with simple logistic regression analyses. Women were divided into two categories according to parity (number of births): primiparous (reference category) and multiparous, i.e. women with two or more births. Maternal and paternal employment status was divided into two categories: employed vs. not employed. Maternal and paternal education was stratified into two levels: higher education (passed at least final high school exams, e.g. upper general or specialized secondary and academic education) and lower education (secondary education without final high school exams, such as primary education, lower secondary or basic vocational education). Because employment status and education were strongly related, we calculated an Employment & Education indicator which allowed us to categorize mothers and fathers into four groups: “Not Employed–Poorly Educated”, “Not Employed–Highly Educated”, “Employed–Poorly Educated” and “Employed–Highly Educated”, with the last being the reference group.

We also tested the distribution of characteristics of births among primiparous and multiparous mothers using a t-test for separate variance estimates of independent samples with normal distribution, and a Chi-square test for categorical variables. The distribution of birth characteristics among missing paternal data groups (yes vs. no) was also investigated with the same tests as mentioned above.

After a descriptive analysis of the parental–infant characteristics and their distribution among LBW–NBW children among the strata of parity and among the missing paternal data groups, we performed simple and multiple logistic regression analyses to estimate the association between missing paternal data as a categorical variable and the odds ratio (OR) of LBW. Firstly, only the missing paternal data was entered as a predictor of LBW (crude effect). In the second stage, we built the full model by entering biological factors such as maternal age (continuous), sex of the child, parity, and social factors (Employment & Education groups and marital status) and tested whether there was a significant change in the log odds of LBW after adding these factors to the simple model.

Because the main research objective of our study was to determine if multiparous mothers are more susceptible than primiparous mothers to the effect of missing paternal data (a proxy for paternal investment) on the risk of LBW, we conducted the same procedure after stratification for parity. The influence of parity on the observed association, i.e. effect modification by parity, was further tested by adding the product term (missing paternal data x parity) into the full logistic regression model. The statistical significance of this product term was verified with the Wald test. The interaction (effect modification) related to the state in which the effect of one exposure (missing paternal data) on an outcome (LBW) differs across the strata of another exposure (primiparous and multiparous). The adjusted Synergy Factor [31] were derived from the product term in this multiplicative model. The Synergy Factor (SF), defined as the ratio of the observed OR for both factors combined, to the predicted OR assuming independent effects of each factor; SF> 1 indicates positive interaction (synergy).

## Results

Among the study group of 80 533 births, father's age, education and employment were recorded for N = 77 242 (95.9%), N = 77 071 (95.7%), N = 77 041 (95.6%) neonates (respectively), whilst for N = 3 257 (4,04%) all of the above three categories were left missing.

The univariate analysis suggested that the odds ratio of LBW was significantly associated with paternal and maternal age, parity, sex of the offspring, maternal education, employment status and marital status (Table 1).

**Table 1:** Characteristics associated with LBW among singleton, live, full-term births—results of simple logistic regression analyses presented as odds ratios (OR) with 95% confidence intervals (CI) and p-value.

Characteristics	Level of variable	LBW	NBW	OR	-95% CI	+95% CI	p
Maternal age (cont.)	N	1615	78938	1.00	0.99	1.01	0.82
	Mean	28.2	28.2				
	SD	5.9	5.1				
Paternal age (cont.)	N	1466	75776	1.01	1.00	1.02	0.01
	Mean	31.0	30.6				
	SD	6.5	5.8				
Sex of the child	Female	976	38217	1.63	1.47	1.80	<0.01
	Male	639	40721				[ref.]
Parity	Multiparous	689	36127	0.88	0.80	0.97	0.01
	Primiparous	926	42806				[ref.]
Maternal marital status	Not married	383	10833	1.95	1.74	2.20	<0.01
	Married	1232	68105				[ref.]
Maternal employment status	Employed	1040	58875	0.62	0.56	0.69	<0.01
	Unemployed	560	19812				[ref.]
Maternal education	Higher	1023	61838	0.48	0.44	0.54	<0.01
	Lower	575	16850				[ref.]
Emp & Edu Mother	Not Employed–Poorly Educated	347	7884	2.71	2.38	3.08	<0.01
	Employed–Poorly Educated	227	8949	1.56	1.34	1.81	<0.01
	Not Employed–Highly Educated	212	11913	1.09	0.94	1.28	0.24
	Employed–Highly Educated	811	49891				[ref.]
Paternal employment status	Employed	1213	67231	0.62	0.54	0.71	<0.01
	Unemployed	244	8353				[ref.]
Paternal education	Higher	853	53180	0.59	0.53	0.66	<0.01
	Lower	607	22431				[ref.]
Emp & Edu Father	Not Employed–Poorly Educated	166	4435	2.38	2.00	2.82	<0.01
	Employed–Poorly Educated	438	17967	1.55	1.38	1.74	<0.01
	Not Employed–Highly Educated	78	3901	1.27	1.00	1.61	0.05
	Employed–Highly Educated	775	49224				[ref.]
Missing paternal data	Yes	147	3110	2.44	2.05	2.90	<0.01
	No	1468	75828				[ref.]

Emp & Edu–indicator of employment and education; N- number of participants, SD–standard deviation; LBW-low birth weight (<2500 g), NBW–normal birth weight (≥2500 g)

Mothers who had female newborns had a 63% higher odds for LBW than those who gave birth to males. Each year of paternal age was associated with an increasing OR for LBW by 1%. Unmarried women had a 95% higher odds for delivering an infant with LBW than married women. Among mothers, having a higher education level was associated with 52% lower odds of LBW in comparison to lower education levels, whilst being employed vs. unemployed decrease the odds by 38%. When analyzing maternal educational and employment status together, women who simultaneously had lower education and no employment, had almost three times higher OR for delivering a child with LBW than women with both higher educational level and employment. Similar patterns were seen for paternal education and employment. Taking these two paternal characteristics into account at once, fathers who simultaneously had lower education and no employment, had more than a twofold increase in OR in comparison to those with both higher educational level and employment. Mothers having two or more births in comparison to primiparous mothers had lower odds of LBW by 12% (Table 1). The missing paternal data groups differed according to maternal age, maternal marital, employment and educational status, and Employment & Education indicator calculated for mothers, but not the sex of the child (Table 2).

**Table 2:** Characteristics of singleton, live, full-term births stratified for missing paternal data (a proxy of paternal investment).

Characteristics	Level of variable		missing paternal data		p-value
			(no)	(yes)	
Maternal age	(cont.)	N	77296	3257	<0.001
		Mean	28.3	23.8	
		SD	5.0	6.4	
Sex of the child	Female	N	37630	1563	0.44
		%	96.0%	4.0%	
	Male	N	39666	1694	
		%	95.9%	4.1%	
Maternal marital status	Not married	N	7959	3257	<0.001
		%	71.0%	29.0%	
	Married	N	69337	0	
		%	100.0%	0.0%	
Maternal employment status	Employed	N	58717	1198	<0.001
		%	98.0%	2.0%	
	Unemployed	N	18435	1937	
		%	90.5%	9.5%	
Maternal education	Higher	N	61693	1168	<0.001
		%	98.1%	1.9%	
	Lower	N	15461	1964	
		%	88.7%	11.3%	
Emp & Edu Mother	Not Employed–Poorly Educated	N	6739	1492	<0.001
		%	81.9%	18.1%	
	Employed–Poorly Educated	N	8709	467	
		%	94.9%	5.1%	
	Not Employed–Highly Educated	N	11684	441	
		%	96.4%	3.6%	
	Employed–Highly Educated	N	49976	726	
		%	98.6%	1.4%	

Emp & Edu–indicator of employment and education; N- number of participants, SD–standard deviation; LBW-low birth weight (<2500 g), NBW–normal birth weight (≥2500 g)

The strata of parity varied according to maternal and paternal age, maternal marital, employment and educational status, Employment & Education indicator calculated for mothers, sex of the child, paternal employment and educational status, and Employment & Education indicator calculated for fathers (Table 3).

**Table 3:** Characteristics of singleton, live, full-term births stratified for parity.

Characteristics	Level of variable		Multiparous	Primiparous	p-value
Maternal age	(cont.)	N	36816	43732	<0.001
		Mean	30.6	26.1	
		SD	4.8	4.6	
Paternal age	(cont.)	N	35871	41369	<0.001
		Mean	32.9	28.6	
		SD	5.5	5.3	
Sex of the child	Female	N	18039	21153	0.08
		%	46.0%	54.0%	
	Male	N	18777	22579	
		%	45.4%	54.6%	
Maternal marital status	Not married	N	3680	7533	<0.001
		%	32.8%	67.2%	
	Married	N	33136	36199	
		%	47.8%	52.2%	
Maternal employment status	Employed	N	27055	32857	<0.001
		%	45.2%	54.8%	
	Unemployed	N	9593	10779	
		%	47.1%	52.9%	
Maternal education	Higher	N	27341	35518	<0.001
		%	43.5%	56.5%	
	Lower	N	9314	8110	
		%	53.5%	46.5%	
Emp & Edu Mother	Not Employed–Poorly Educated	N	4324	3907	<0.001
		%	52.5%	47.5%	
	Employed–Poorly Educated	N	4978	4197	
		%	54.3%	45.7%	
	Not Employed–Highly Educated	N	5264	6861	
		%	43.4%	56.6%	
	Employed–Highly Educated	N	22065	28635	
		%	43.5%	56.5%	
Paternal employment status	Employed	N	32138	36304	<0.001
		%	47.0%	53.0%	
	Unemployed	N	3592	5005	
		%	41.8%	58.2%	
Paternal education	Higher	N	23685	30346	<0.001
		%	43.8%	56.2%	
	Lower	N	12071	10967	
		%	52.4%	47.6%	
Emp & Edu Father	Not Employed–Poorly Educated	N	2309	2292	<0.001
		%	50.2%	49.8%	
	Employed–Poorly Educated	N	9741	8664	
		%	52.9%	47.1%	
	Not Employed–Highly Educated	N	1276	2703	
		%	32.1%	67.9%	
	Employed–Highly Educated	N	22382	27615	
		%	44.8%	55.2%	
Missing paternal data	No	N	35890	41404	<0.001
		%	46.4%	53.6%	
	Yes	N	926	2328	
		%	28.5%	71.5%	

Emp & Edu–indicator of employment and education; N- number of participants, SD–standard deviation; LBW-low birth weight (<2500 g), NBW–normal birth weight (≥2500 g)

**Fig 1:**
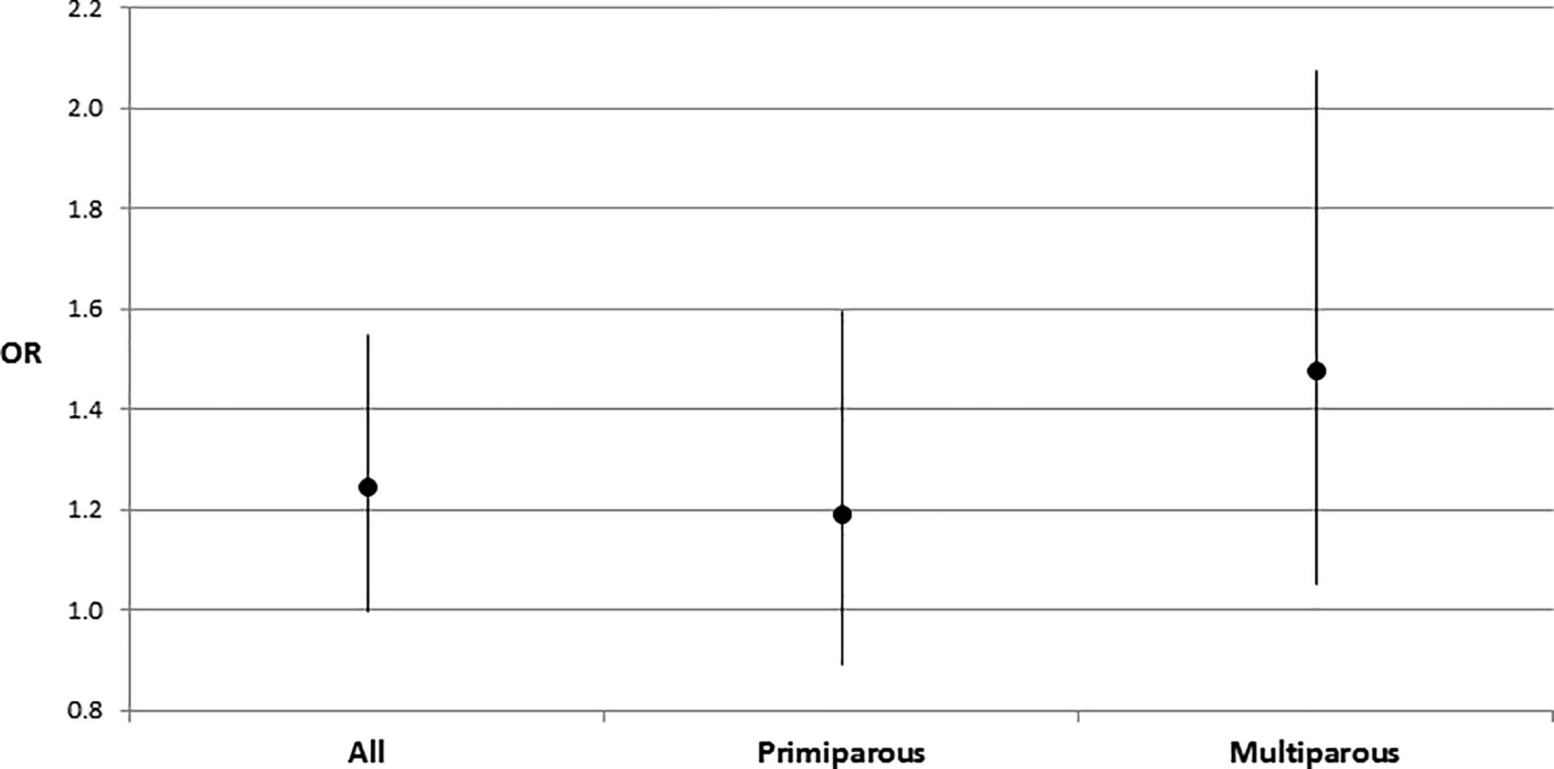
Adjusted odds ratios with 95% confidence intervals for the association between the lack of paternal investment (measured as missing paternal data in birth records) and low birth weight across the strata of parity. All presented effects are standardized to confounders. Effects for primiparous and multiparous mothers come from models estimated using only those subgroups.

The univariate analyses revealed that missing paternal data were associated with almost 2.5 times increased odds for LBW in comparison to paternal data present (OR = 2,44; 95% CI 2.05–2.90). After standardization to maternal-infant characteristics, this association was weaker, and marginally significant (OR = 1.24; 95% CI 1.00–1.55). After stratification for parity, elevated odds of LBW noted among primiparous mothers in crude analyses (OR = 1.7; 95% CI 1.37–2.18) did not reach the level of statistical significance after adjustment (OR = 1.19; 95% CI 0.89–1.59). However, among the strata of multiparous mothers, missing paternal data was associated with higher odds of LBW both before (OR = 4.34; 95% CI 3.34–5.65) and after adjustment for confounders (OR = 1.48; 95% CI 1.05–2.08). Strata specific estimates after standardized to confounders are presented in Table 4. The association between the missing paternal data and low birth weight among the entire group and across the strata of parity, after standardization to confounders, is plotted on Fig 1.

**Table 4:** Modification of the effect of missing paternal data (a proxy of paternal investment) on LBW by parity categories (primiparous v. multiparous).

	Missing paternal data (No)		Missing paternal data (Yes)		
	N with LBW/ NBW	OR (95% CI)	N with LBW/ NBW	OR (95% CI)	OR (95% CI) for missing paternal data (yes) vs. Missing paternal data (no) within strata of parity
Primiparous	844/40481	1.00 (reference)	78/2197	0.96 (0.73–1.25); p = 0.74	1.19 (0.89–1.59); p = 0.24
Multiparous	618/35163	0.65 (0.57–0.73); p<0.001	57/793	1.32 (0.97–1.80); p = 0.08	1.48 (1.05–2.08); p = 0.02

Synergy Factor (SF) = 2.12 (95% CI 1.47–3.05, p<0.001)

ORs are adjusted for marital status, Emp & Edu, sex of the child and maternal age

These different effects of missing paternal data across the subgroups of parity (OR = 1.19; 95% CI 0.89–1.59 among primiparous, and OR = 1.48; 95% CI 1.05–2.08 among multiparous) were confirmed by the statistically significant product term in logistic regression model after standardizing to confounders (Wald test, p<0.001).

In the second step of our analysis of interaction we calculated four OR’s, that is those associated with each factor in the presence or absence of the other factor, with taking subjects with neither factor as the baseline (Table 4). Thus, taking primiparous mothers with paternal data present as the reference, the OR for being multiparous alone was 0.65 (95% CI 0.57–0.73), whilst that for missing paternal data was 0.96 (95% CI 0.73–1.25). That gave a predicted OR of 0.62 (= 0.65 x 0.96) for the combination of both factors, compared with an observed OR of 1.32 (95% CI 0.97–1.80). Hence, the Synergy Factor = 2.12 (95% CI 1.47–3.05, p<0.001). Thus, the null hypothesis of no interaction was rejected and significant synergy was found. The observed joint effect of the two factors was above two times greater than the predicted joint effect.

## Discussion

We showed that maternal reproductive effort during gestation, manifested in an infant’s birth weight, may be sensitive to environmental conditions such as lack of paternal investment and number of existing children. We found that a lack of paternal investment (indicted by missing paternal data in birth records) was associated with higher odds of LBW. This relationship was statistically significant even after standardization to factors with a well-established impact on birth weight (i.e. maternal age, marital status, Employment & Education indicator, parity and sex of the child). Stratified analysis revealed that the effect of missing paternal data on the odds of LBW differed according to parity. On the basis of the stratum-specific estimates, missing paternal data was more strongly associated with LBW in multiparous than in primiparous mothers. After adjustment for confounders among the strata of multiparous mothers, missing paternal data was associated with higher odds of LBW (1.48; 95% CI 1.05–2.08), whilst the same association was not statistically significant among primiparous women (p = 0.24). The effect modification by parity was confirmed by the test for interaction on the multiplicative scale (Wald test, p<0.001). The study has shown that missing paternal data and multiparity interact in evoking the low birth weight. We found that the combination of missing paternal data and being multiparous increased the odds of low birth weight in comparison to expected diminishing of risk from the product of the individual effects of these two exposures. Our results suggest that multiparous mothers may be more vulnerable to little or no paternal investment than primiparous mothers; therefore we propose treating parity as a potential modifier of the association between paternal investment and the risk of LBW.

There are no empirical studies, to our knowledge, which show the association between a partner’s support and the risk of LBW with respect to parity. However, making reference to life history theory, it is likely that the decisions mothers face between investment in current vs. future reproduction may be affected by the number of dependent children they already have. High parity may serve as a proxy for high costs of reproduction [8, 20] and concomitant low level or lack of partner support could result in reduced investment in current offspring, manifested as infants with low birth weight.

The unfavorable environment that exists when a father provides little or no support and previous children still require attention and care make women follow a reproductive strategy that results in lower investment in the current pregnancy, which is expressed in reduced fetal growth. Such a strategy may be viewed as a response to ecological circumstances, shaped through phenotypic plasticity. We assumed that physiological responses to little or no paternal investment may be guided by evolved mechanisms and can be predicted by evolutionary theories. Comparative studies indicate that human offspring are costly to produce, mature slowly, and reach nutritional independence late [32]. These features imply demanding parental care requirements, which can rarely be supplied by the mother alone [32–34]. The cooperative breeding hypothesis states that allomaternal assistance must have been essential for child survival during our evolutionary past [33,35]. It is suggested that significant paternal investment was important for the evolution of Homo sapiens and mothers who had such support had a selective advantage in producing and raising offspring to reproductive maturity compared with mothers who did so alone [35–36]. For human mothers, both fathers and alloparents helped to bear the costs of childcare, which probably contributed to evolving (as is unusual among other species) life history capacity–simultaneously taking care of many dependent children who require a lot of investment [37–38]. It is still unclear how important paternal investment was in our evolutionary history [39]; however, evidence across studies suggests that the impact of a partner’s support is more beneficial to the health of pregnant women and her offspring than the role of any other member of a woman’s social network [40]. Firstly, a partner’s support may encourage a pregnant woman to practice healthy pregnancy behaviours and introduce lifestyle changes that can improve her physical health and thus the health of the offspring [41]. Namely, women whose partners are involved in their pregnancy are more likely to receive early prenatal care [15] and reduce cigarette and alcohol consumption over the course of the pregnancy [15,17,42]. Secondly, the emotional support and material resources provided by the father may mitigate the physical and psychological strains associated with pregnancy [40]. It was found that effective (i.e. to the extent which meets the mother’s needs) support from a partner correlated well with lower anxiety in mid-pregnancy and reduction in anxiety from mid to late pregnancy [43]. It is well established that general maternal distress and depression during pregnancy negatively influence fetal growth and prospectively predict adverse perinatal outcomes, including reduced infant birth weight [44–45].

There are multiple routes by which paternal support may be linked to variation in the birth weight of offspring. A lack of paternal support may induce psychological stress in the mother, which can lead to altered fetal growth through neural and endocrine pathways [46]. Maternal stress can act through the hypothalamic–pituitary–adrenal axis, which is modulated by corticotropin-releasing hormone [47–48]. Moreover, psychosocial stress during pregnancy may also affect immune functioning. Placental production of corticotropin-releasing hormone (which may be activated by maternal stress) can lead to the release of pro-inflammatory cytokines, whilst elevated inflammation can result in adverse birth outcomes [49–51]. Women who had some support had lower CRP levels during the third trimester of pregnancy [49]. It should be noted, however, that findings related to the inflammatory mechanisms linking social support and birth outcomes are not consistent in the literature [52].

Another mechanism through which parity can be linked to birth outcomes is related to maternal supply of nutrients to the fetus. The risk of folic acid deficiency [53] and lower deposits of (n-3) fatty acids in maternal plasma phospholipids [54] is especially pronounced among multiparous in comparison to primiparous mothers. It is plausible that an unfavourable environment, including poor maternal diet and closely spaced births [22], concomitant with low social support may affect infants’ birth weight. All of these observations suggest the existence of biological mechanisms that may influence interactions between low paternal investment and parity and, in consequence, may explain variations in neonatal birth weight.

Like other studies based on birth registries, our research has several limitations, one of which is that the type of paternal involvement and partner relationship quality cannot be examined in detail. In our study we used a proxy indicator of paternal investment; however, similar indicators were also used in other studies based on birth certificates [26–29,41]. We acknowledge that missing paternal data should be treated as an imperfect measure of paternal investment. It is likely that some men whose data are listed on birth certificates are nonetheless low investors. They may abandon the mother soon after birth and, even if they remain with the mother, they do not necessarily invest in the offspring. Additionally, paternal data might be missing for reasons unrelated to low paternal investment (for example, in the case of women who used donor insemination).

According to Polish law, for married couples the husband of the child's mother is usually assumed to be the father of the child. Therefore, in our data there were no married mothers with missing paternal data. When a child's parents are not married, recognition (acknowledgment) of paternity by the father is required, therefore missing paternal data is noted only for unmarried mothers. Such an observation raised the problem of multicollinearity between marital status and missing paternal data. In order to verify this, we used the variance inflation factor (VIF). We found that VIF for missing paternal data equaled 1.4, which indicates a small correlation between this variable and all others in the model. The same value was found for marital status (VIF = 1.4). According to the literature, VIF should not exceed 10, while in logistic regression models, values above 2.5 may be a cause for concern [55]. Thus, our calculations suggest that there is no problem with multicollinearity in this study.

We added further support to the hypothesis that a low level of support from a partner may affect a woman’s trade-offs between investment in current vs. future reproduction [7, 12]. We also defined and confirmed our new hypothesis that the negative impact of low paternal support may differ in relation to the parity status of the mother. Women who already have children may have a different resource allocation strategy than women giving birth to their first child. For mothers facing environmental stress caused by a lack of partner investment it may be more beneficial to invest more in the children they already have, rather than in the current pregnancy. Older children have higher reproductive value and such a strategy may thus benefit maternal fitness [56].

Further studies are needed to verify these predictions. Taking into account recent changes in family structure (an increasing number of children are born outside of marriage [57]) even in Catholic countries such as Poland [58] the impact of paternal involvement on maternal health and pregnancy outcomes needs to be further explored. Instability in the psycho-social environment during the gestational stage of development affects not only the health of offspring at delivery and in infancy, but also has lifelong consequences for the child because LBW neonates have a higher risk of metabolic diseases (among others) in future life [59]. From this point of view, our results pave the way for additional research into support intervention. Regarding recommendations for future studies, it would be interesting to evaluate the effect of alloparents on pregnancy investment, to assess whether paternal investment is still important despite potential high levels of alloparental care. Likewise, it would be intriguing to determine whether the relationships we have found are affected by paternity certainty, the social status of the father, and female features such as: attractiveness, parenting skills and strategies to induce paternal investment [12].
